# Tryptophan metabolism and disposition in cancer biology and immunotherapy

**DOI:** 10.1042/BSR20221682

**Published:** 2022-11-11

**Authors:** Abdulla A.-B. Badawy

**Affiliations:** Formerly School of Health Sciences, Cardiff Metropolitan University, Western Avenue, Cardiff CF5 2YB, Wales, U.K.

**Keywords:** 3-dioxygenase, 3dioxygenase, Aryl hydrocarbon receptor, indoleamine 2, kynurenic acid, kynurenine pathway, tryptophan 2, tumor immune escape

## Abstract

Tumours utilise tryptophan (Trp) and its metabolites to promote their growth and evade host defences. They recruit Trp through up-regulation of Trp transporters, and up-regulate key enzymes of Trp degradation and down-regulate others. Thus, Trp 2,3-dioxygenase (TDO2), indoleamine 2,3-dioxygenase 1 (IDO1), IDO2, N′-formylkynurenine formamidase (FAMID) and Kyn aminotransferase 1 (KAT1) are all up-regulated in many cancer types, whereas Kyn monooxygenase (KMO), kynureninase (KYNU), 2-amino-3-carboxymuconic acid-6-semialdehyde decarboxylase (ACMSD) and quinolinate phosphoribosyltransferase (QPRT) are up-regulated in a few, but down-regulated in many, cancers. This results in accumulation of the aryl hydrocarbon receptor (AhR) ligand kynurenic acid and in depriving the host of NAD^+^ by blocking its synthesis from quinolinic acid. The host loses more NAD^+^ by up-regulation of the NAD^+^-consuming poly (ADP-ribose) polymerases (PARPs) and the protein acetylaters SIRTs. The nicotinamide arising from PARP and SIRT activation can be recycled in tumours to NAD^+^ by the up-regulated key enzymes of the salvage pathway. Up-regulation of the Trp transporters SLC1A5 and SLC7A5 is associated mostly with that of TDO2 = FAMID > KAT1 > IDO2 > IDO1. Tumours down-regulate enzymes of serotonin synthesis, thereby removing competition for Trp from the serotonin pathway. Strategies for combating tumoral immune escape could involve inhibition of Trp transport into tumours, inhibition of TDO and IDOs, inhibition of FAMID, inhibition of KAT and KYNU, inhibition of NMPRT and NMNAT, inhibition of the AhR, IL-4I1, PARPs and SIRTs, and by decreasing plasma free Trp availability to tumours by albumin infusion or antilipolytic agents and inhibition of glucocorticoid induction of TDO by glucocorticoid antagonism.

## Introduction

Metabolism of the essential amino acid *L*-tryptophan (Trp) has gained prominence in cancer research and immunotherapy for four main reasons: (1) the expression of the Trp-degrading enzymes hepatic Trp 2,3-dioxygenase (TDO, formerly Trp pyrrolase; EC 1.13.11.11) and extrahepatic indoleamine 2,3-dioxygenase (IDO; EC 1.13.11.17) in tumours; (2) the production of immunomodulatory kynurenine (Kyn) metabolites in the Kyn pathway (KP) of Trp degradation, some of which are proinflammatory; (3) the use of these proinflammatory metabolites by tumours to suppress innate immunity; (4) the control of tumoral [Trp] by tumours to their advantage and to undermine immune defences, thereby achieving their immune escape. In this review, Trp metabolism will be discussed in some detail, with emphasis on enzymes and metabolites of the KP. Although enzymes and metabolites of Trp metabolism exert a range of effects on, and are influenced by, various body systems [1], emphasis here will focus on their effects on the immune system, which may directly or indirectly impact tumour biology and therapeutic strategies. The disposition of Trp as an essential nutrient in both host and tumour, the ability of tumours to ensure an adequate supply of Trp for their own proliferation and survival, but low enough to deprive T cells will be discussed and means of combating the tumour by undermining its Trp-directed actions will be suggested. As much information on the biology of cancer in relation to Trp metabolism and related biological processes has already been established, the present text will not be exhaustive, but focused mainly on specific issues relevant to the above title.

## Overview of tryptophan metabolism

### Introductory overview

Tryptophan (Trp) is an essential amino acid and is thus vital for protein synthesis. As very little dietary Trp, if at all, is used for protein synthesis under normal steady-state conditions of net nitrogen balance, wherein the Trp released from protein breakdown is reutilised for protein synthesis [2], the bulk of dietary Trp is available for metabolism. Trp is metabolised in mammals via 4 known pathways, three of which, the transamination, decarboxylation and hydroxylation pathways are of minor quantitative, but substantial functional, significance. Each of these 3 pathways contributes ≤ 1% to Trp degradation. The fourth pathway, the KP, accounts for ∼95% of Trp degradation [2–4] and produces a wide range of physiologically active intermediates that influence many body functions and play important roles in health and disease. Gut microbiota is another source of Trp metabolites, mainly indole derivatives, but can also synthesise Trp. In the following text, brief accounts of these 4 pathways will precede the bulk of this review.

### The transamination pathway

**Figure 1:**
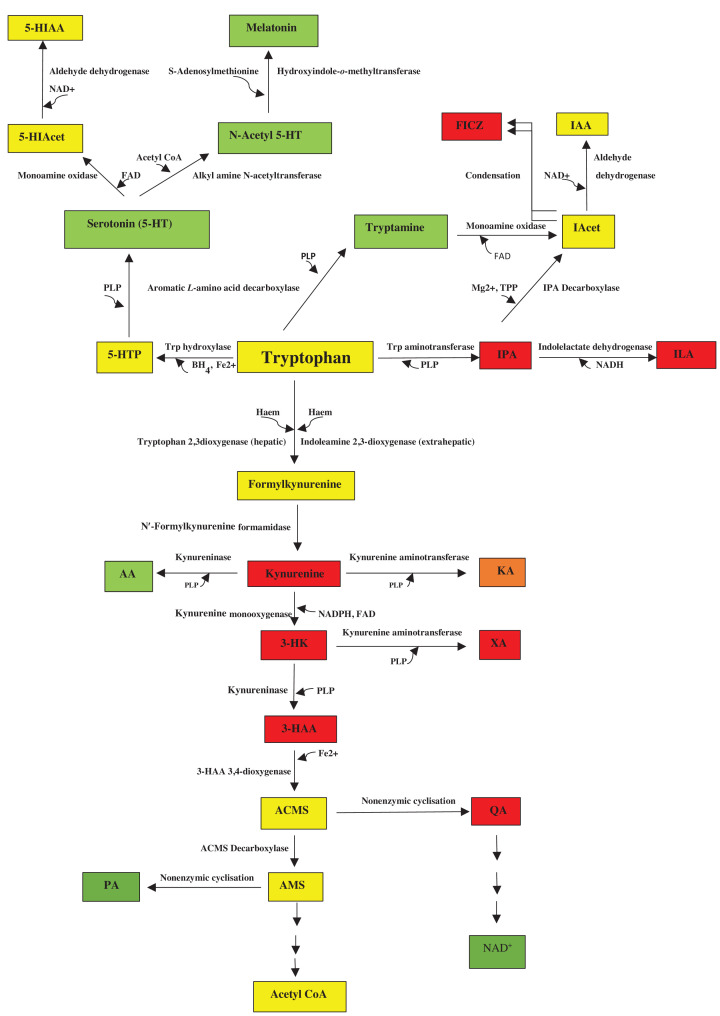
The tryptophan degradative pathways This figure is adapted from Figure 1 in [5] by Badawy, A.A.-B. and Guillemin, GJ. Species differences in tryptophan metabolism and disposition. *Int. J. Tryptophan. Res*. 2022, **15**, 1-26). Abbreviations: 3-HAA, 3-hydroxyanthranilic acid; 3-HK, 3-hydroxykynurenine; 5-HIAA, 5-hydroxyindoleacetic acid; 5-HIAcet, 5-hydroxyindoleacetaldehyde; 5-HT, 5-hydroxytryptamine or serotonin; 5-HTP, 5-hydroxytryptophan; AA, anthranilic acid; Acet, acetaldehyde; ACMS, 2-amino-3-carboxymuconic acid-6-semialdehyde: also known as acroleyl aminofumarate; AMS, 2-aminomuconic acid-6-semialdehyde; IAcet, indole acetaldehyde; IAA, indol-3-ylacetic acid; ILA, indol-3-yllactic acid; IPA, indol-3-ylpyruvic acid; KA, kynurenic acid; PA, picolinic acid; QA, quinolinic acid; XA, xanthurenic acid. Colour code: red (proinflammatory), green (antiinflammatory), amber (dually acting) and yellow (normal metabolite).

Trp is transaminated to IPA via an unstable intermediate by the action of Trp aminotransferase (Figure 1) [5]. As well as from Kyn in the KP, kynurenic acid (KA) can also be formed from IPA via the unstable Kynuric acid intermediate formed with participation of reactive oxygen species (ROS). Although ∼60% of Trp transamination in rat liver homogenates is due to tyrosine-2-oxoglutarate aminotransferase [6], several aminotransferases specific for Trp exist in rats, bacteria and plants, using various co-substrates [7]. Transamination of Trp to IPA is reversible, at least by gut microbiota, which may explain the ability of exogenous IPA to restore nitrogen balance in a healthy woman maintained on a synthetic Trp-free diet [8]. Previously, it was shown [9] that Trp-deficient rats gained weight after dietary supplementation with IPA. As will be described below, IPA is further metabolised by gut microbiota to other biologically active metabolites.

### The decarboxylation pathway

Trp is decarboxylated to tryptamine. Tryptamine undergoes oxidative deamination to indol-3-ylacetaldehyde, which is then oxidised to indol-3-ylacetic acid. As will be stated below, tryptamine is also formed from Trp by gut microbiota. Figure 1 also shows the metabolic formation of the biologically active compound FICZ (6-formylindolo [3,2-b]carbazole), originally identified as a product of Trp photo-oxidation, achieved by condensation of two molecules of indol-3-ylacetaldehyde or at least one molecule plus the α-hydroxylated metabolite of the other [10].

### Tryptophan metabolism by gut microbiota

**Figure 2:**
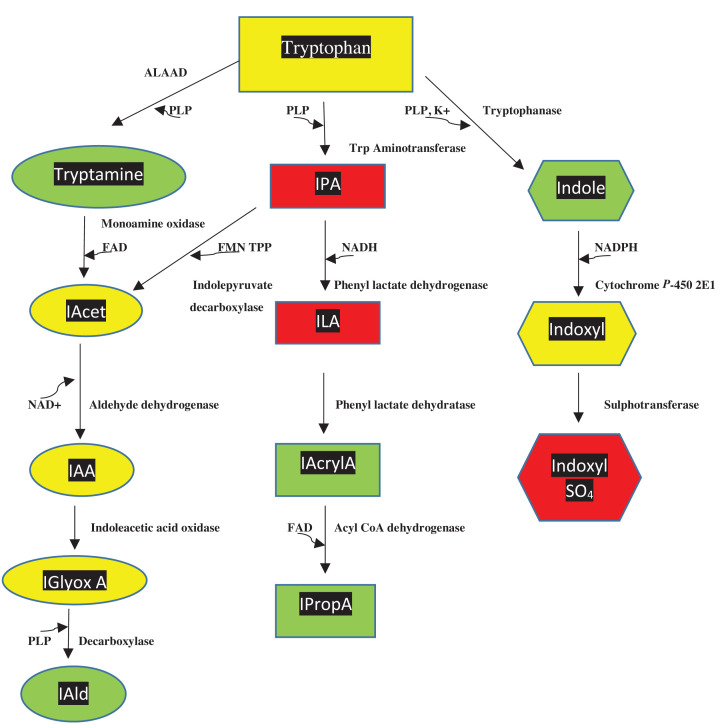
Tryptophan metabolism by gut microbiota Abbreviations: ALAAD, aromatic *L*-amino acid decarboxylase; FAD, flavin-adenine dinucleotide; FMN, flavin mononucleotide; IAA, indoleacetic acid; IAcet, indole acetaldehyde; IAcrylA, indole acrylic acid; IAld, indole aldehyde; IGlyoxA, indole glyoxylic acid; ILA, indole lactic acid; IPA, indole pyruvic acid; IpropA, indole propionic acid; NAD(P)H, reduced nicotinamide-adenine dinucleotide (phosphate); PLP, pyridoxal 5′-phosphate. Colour code: as in Figure 1.

The gut microbiota plays a significant role in Trp metabolism, specialising in production of indoles [11] (Figure 2). They metabolise Trp to indole by tryptophanase, to tryptamine by ALAAD and to IPA by Trp aminotransferase. Brain and cerebrospinal fluid tryptamine levels are partly derived from the periphery, whereas those of indol-3-ylpropionic acid (IPrA) are derived solely from the periphery [12]. Two gut bacteria, the common gut firmicutes *Clostridium sporogenes* and *Ruminococcus gnavus*, encode Trp decarboxylase (ALAAD) in ∼10% of the human population [13]. As will be described below, many of the Trp and indole metabolites produced by gut microbiota possess important biological properties.

### The hydroxylation pathway

Serotonin synthesis is a 2-step process in which Trp is first hydroxylated to 5-hydroxytryptophan (5-HTP), which is then decarboxylated to serotonin (5-HT). Because Trp hydroxylase TPH is partially (≤50%) saturated with its Trp substrate [14], its activity is largely dependent on [Trp] both in the periphery and the brain. Additionally, cerebral serotonin synthesis is dependent on plasma Trp availability to the brain, which is determined by (1) activity of hepatic TDO; (2) plasma albumin binding of Trp; and (3) extent of competition with Trp for entry into the brain by at least 5 competing amino acids (CAA), namely Val, Leu, Ile, Phe and Tyr [15]. As well as by TPH2, human brain serotonin synthesis can also be controlled by the pyridoxal 5′-phosphate (PLP)-dependent aromatic *L*-amino acid decarboxylase (ALAAD), whose activity is low [16] and can be impaired in nutritional vitamin B_6_ deficiency or upon inactivation of its PLP cofactor by drugs. As will be discussed below, tumours have their own mechanisms of acquiring Trp. Serotonin is oxidatively deaminated to 5-hydroxyindoleacetaldehyde, which is then oxidised to 5-hydroxyindoleacetic acid. In both pineal and the periphery, 5-HT is converted to melatonin in a 2-step process, the first of which is the rate-limiting. Approximately 90% of serotonin is synthesised in the periphery via the sequential catalytic actions of the TPH isoform 1 (TPH1) and ALAAD, mainly in enterochromaffin cells. TPH1 activity is modulated by intestinal microbiota, which enhance TPH1 mRNA expression [17] through short-chain fatty acids [18].

### The oxidative (kynurenine) pathway

Here, Trp is first oxidised to N′-formylkynurenine by either Trp 2,3-dioxygenase (TDO) mainly in liver or indoleamine 2,3-dioxygenase (IDO) elsewhere including immune cells. The abundant N′-formylkynurenine formamidase (FAMID) hydrolyses N′-formylkynurenine to kynurenine (Kyn). Kyn is metabolised mainly by oxidation to 3-hydroxykynurenine (3-HK) by Kyn monooxygenase (KMO: also known as Kyn hydroxylase) followed by hydrolysis of 3-HK to 3-hydroxyanthranilic acid (3-HAA) by kynureninase (KYNU). This latter enzyme can also hydrolyse Kyn to anthranilic acid (AA). 3-HAA is oxidised by 3-HAA 3,4-dioxygenase (3-HAAO) to 2-amino-3-carboxymuconic acid-6-semialdehyde (ACMS: also known as acroleyl aminofumarate). ACMS occupies a central position at two junctions of the KP. The pathway favours its non-enzymic cyclisation to quinolinic acid (QA) and subsequent *de novo* synthesis of NAD^+^. The second junction involves decarboxylation of ACMS by ACMS decarboxylase (ACMSD: also known as picolinate carboxylase) to 2-amino-3-muconic acid-6-semialdehyde (AMS). AMS can also cyclise non-enzymically to picolinic acid (PA), but only if AMS dehydrogenase is substrate-saturated, otherwise it proceeds to eventual formation of acetyl CoA.

**Figure 3:**
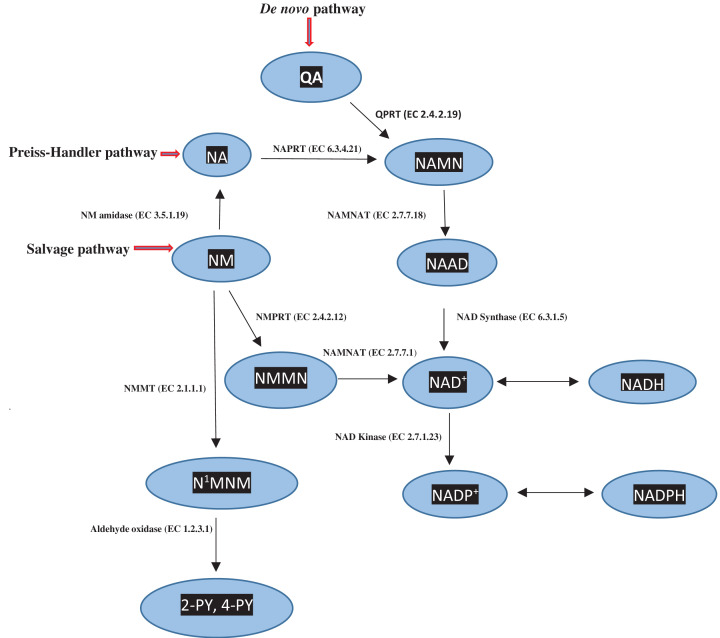
NAD^+^ synthesis from the *de novo*, Preiss-Handler and Salvage pathways Adapted here from Figure 2 in [3] by Badawy, A.A.-B. (2017) Kynurenine pathway of tryptophan metabolism: regulatory and functional aspects. *Int J Tryptophan Res*. **10**, 1–20, doi: 10.1177/1178646917691938. Abbreviations: NA, nicotinic acid; NAAD, nicotinic acid-adenine-dinucleotide; NAD^+^(P^+^)H, oxidized and reduced nicotinamide-adenine dinucleotides (phosphates); NAMN, nicotinic acid mononucleotide; NAMNAT, nicotinic acid mononucleotide adenylyltransferase; NAPRT, nicotinic acid phosphoribosyltransferase; N^1^MNM, N^1^-methylnicotinamide; NM, nicotinamide; NMMN, nicotinamide mononucleotide; NMNAT, nicotinamide mononucleotide adenylyltransferase; NMPRT, nicotinamide phosphoribosyltransferase; 2- and 4-PY, 2- and 4-pyridone carboxamides; QA, quinolinic acid.

Figure 3 illustrates NAD^+^ synthesis in the *de novo* pathway from QA, the Preiss-Handler pathway from nicotinic acid and the salvage pathway from nicotinamide. In humans and many other mammals, the KP favours NAD^+^ synthesis from Trp in the *de novo* biosynthetic pathway, rather than via the other two pathways. The mechanisms underpinning the poor contribution of the latter pathways to NAD^+^ synthesis were elucidated by the elegant enzymatic studies of the group of D A Bender (see [3] for details and references). One such key enzyme is ACMSD whose activity determines how much ACMS is cyclised to QA or diverted towards picolinic acid (PA) formation. For example, the very high ACMSD activity of the domestic cat (*Felis catus*), compared with that of the rat, limits the ability of the former species to synthesise NAD^+^ from Trp [19]. Gut microbiota, however, play an important role in NAD^+^ synthesis from the Preiss-Handler pathway. Thus, microbiota contribute to the host’s NAD^+^ content by deamidating nicotinamide or its riboside [20].

## Tryptophan disposition

Plasma Trp exists largely bound to albumin, with ∼ 5–10% freely circulating. Although tissues take up only free Trp, the rapid equilibration between the free and bound fractions justifies the need to measure both free and total (free + albumin-bound) Trp. This is particularly important not only in interpreting changes in Trp disposition induced by experimental conditions or disease states, but also to establish the baseline Trp status. Table 1 summarises conditions associated with changes in plasma free and total [Trp] and Trp binding to albumin, expressed as the % free Trp [100 X [free Trp]/[total Trp]). A decrease in plasma total Trp does not automatically reflect enhanced degradation by TDO or IDO but can occur after strong and sustained displacement of bound Trp by direct displacers such as salicylate, clofibrate, other displacing drugs or nonesterified fatty acids (NEFA) following enhanced lipolysis by agents such as catecholamines or methylxanthines, or if plasma [albumin] is decreased, e.g. in pregnancy, liver and kidney diseases, cancer and infections. Under these conditions, (free) Trp availability to tissues is in fact increased and a conclusion of Trp depletion based on a decrease in [total Trp] can therefore be excluded. Circulating free [Trp] is subject to many modulators: physiological, nutritional, pharmacological and methodological, which should be considered in interpreting changes in Trp disposition (for detailed discussion, see [21]). The flux of plasma free Trp through TDO and down the KP, in the absence of changes in TDO (or IDO) activity, is an important determinant of production of KP metabolites and is influenced primarily by changes in circulating albumin and NEFA, both of which undergo significant changes in disease states including cancer (see below). These aspects emphasize the importance of measuring plasma free [Trp], a parameter often neglected in most studies.

**Table 1:** Conditions associated with altered plasma tryptophan concentrations and albumin binding

	Free	Total	% Free
Effectors and examples	Trp	Trp	Trp
Decreased albumin (pregnancy, liver disease, cancer, infection)	↑	-	↑
Decreased NEFA (insulin and other antilipolytic agents)	↓	-	↓
Increased [NEFA] (Lipolysis: catecholamines, ethanol, methylxanthines)	↑	-	↑
Direct Trp displacement (salicylate, clofibrate)	↑	-	↑
Strong and sustained displacement	↑↑	↓	↑↑
TDO induction (glucocorticoids)	↓	↓	-
IDO induction* (cytokines)	?	↓	?
TDO inhibition (antidepressants)	↑	↑	-
IDO inhibition*	?	?	?

Symbols used: ↑ an increase; ↓ a decrease; - a no change; ? unknown effect. With IDO induction*, the decrease in total [Trp] occurs only under conditions of immune activation. With IDO inhibition*, a likely increase in free or total Trp will be limited to reversal of decreases induced by inflammation.

## Immune and other functions of tryptophan metabolites

Of the many functions of Trp metabolites, those related to the immune system are the most relevant to immune diseases including cancer. Important modulators of immune function are the aryl hydrocarbon receptor (AhR), the family of poly (ADP-ribose) polymerases (PARPs) and the protein acetylaters, the sirtuins, e.g. SIRT1 (silent mating type information regulation 2 homologue 1). A brief account of these immune modulators may be useful for the subsequent discussion.

### The aryl hydrocarbon receptor (AhR) and its activation by tryptophan metabolites

The AhR is a transcription factor, activation of which by ligands elicits both destructive and protective effects, with exogenous ligands such as TCDD (2,3,7,8-tetrachlorodibenzo-*p*-dioxin), halogenated hydrocarbons, benz-[*a*]-pyrene and other compounds promoting inflammatory responses, and endogenous ligands counteracting inflammation [22]. Excessive activation by endogenous ligands can however cause harm. Several Trp metabolites are among the endogenous AhR ligands. KA [23] and Kyn [24] were the first reported KP AhR ligands. Of Kyn metabolites, KA is the strongest activator of the AhR, causing levels of activation at 10 µM similar to those induced by a 10 nM concentration of the potent high-affinity ligand TCDD [23]. The other KP metabolite to cause a significant AhR activation at 10 µM is XA but only at 17% of the KA effect. No significant activation is observed with Kyn, 3-HK, AA or QA at 10 µM [23]. The [Kyn] of ≥ 50 µM in the other study [24] is likely be achieved only in ‘closed’ *in vitro* culture systems, rather than in *in vivo* situations or under human pathological conditions. The possibility cannot however be dismissed that at such relatively high [Kyn], KA could be formed, e.g., in glioma cells [25] by Kyn aminotransferase (KAT), whose activity depends on [Kyn] in view of its high *K*_m_ value (0.96–4.7 mM) for the Kyn substrate [3]. At the lower concentration of 100 nM that is ∼3-fold higher than its physiological plasma concentration, KA can contribute 25% of the level of activation exerted by 10 nM TCDD [23]. A 100 nM [KA] can be reached easily if [Kyn] is elevated, as occurs in humans by loading with a relatively small dose of Trp (1.15 g) leading to a 50% increase in plasma [Kyn] and a 3-fold elevation of [KA] [26], or by inhibition of KMO activity, as reported in mice, by *m*-nitrobenzoylalanine raising blood [KA] to 400 nM [27]. The role of KA in AhR activation is further suggested by the observation [28] that the novel metabolic immune check-point: IL-4-induced gene 1 (IL4I1 or *L*-phenylalanine oxidase) activates the AhR via KA (and also IPA). Trp metabolites from other degradative pathways also act as AhR ligands, in particular indole metabolites formed by gut microbiota. In fact, IPA is a stronger AhR ligand than KA [28]. Other indoles are also AhR ligands, including indole itself, indol-3-ylacetic acid, indol-3-yllactic acid, indol-3-ylpropionic acid, indol-3-ylaldehyde and indoxyl sulfate [29–33].

An important aspect of AhR activity in relation to Trp metabolism is its control of IDO1 expression via an autocrine loop involving AhR-IL-6-STAT3 signaling [34,35] and while IL-6 production can be induced by KA, generation of this interleukin by inflammation can induce IDO to produce enough KA to activate the AhR [23]. Another important function of the AhR is control of expression of poly (ADP-ribose) polymerases (PARPs) [28,36], as discussed below.

### Poly (ADP-ribose) polymerases (PARPs) and other NAD^+^-consuming enzymes

A family of enzymes, the PARPs catalyse the transfer of adenosine diphosphate (ADP)-ribose to target proteins, thereby influencing many important processes, including DNA repair. PARP 1 is the most abundant among the PARPs. PARPs are NAD^+^ consumers and activation of the PARP reaction can therefore result in depletion of NAD^+^ and hence also of ATP. It is in part through this depletion that PARP1 over-activation can result in a multifaceted programmed cell death pathway.

Of the PARP family members, PARP 1 is the most active. PARPs 1, 2, 5a and 5b are PARylating (transferring more than two poly ADP-ribose moieties), whereas PARPs 3, 4, 6–12 and 14–16 are Marylating (i.e. transferring only a mono ADP-ribose moiety) and also known as non-canonical PARPs [37]. Activation of the AhR induces up-regulation of these non-canonical PARPs [36], hence they are also referred to as TiPARPs (i.e. TCDD-inducible PARPs). Heer et al. [38] demonstrated up-regulation of many Marylating PARPs by coronavirus SARS-2 and the ability of PARP 10 activation alone to induce NAD^+^ depletion. Moreover, COVID-19 infection (of ferrets) *in vivo* down-regulates NAD^+^ synthesis from Trp in the main *de novo* pathway and from nicotinic acid in the Preiss-Handler pathway but up-regulates that from nicotinamide or its riboside in the ‘salvage’ pathway. As will be described below, this action of the SARS-2 virus is similar to that of tumours and demonstrates the cleverness of these noxious infections in blocking the major and most effective source of NAD^+^ synthesis to the detriment of the host, while promoting activity of the ‘salvage’ pathway to maintain their NAD^+^ metabolome. Another parallel between SARS-2 and cancer is NMNAT up-regulation [38] (see below), which can lead to PARP 1 activation independently of NAD^+^ synthesis [39].

Other NAD^+^-consuming enzymes include sirtuins, NAD^+^ glycohydrolase (NADase), CD38, SARM1 and NAD^+^ kinase. Sirtuins acetylate proteins (e.g. SIRT1) or promote their mono ADP ribosylation (e.g. SIRTs 4 and 6). SARM1 possesses both NAD^+^ glycohydrolase and ADP-ribosyl cyclase activities. NAD kinase phosphorylates NAD^+^ to the other important redox cofactor NADP^+^ [40]. PARP1 and SIRT1 are the major NAD^+^ consumers, each using 33% of cellular NAD^+^, based on inhibitor studies under basal conditions [37]. With DNA damage, e.g., by inflammatory cytokines acting through reactive oxygen (ROS) and/or nitrogen (RNS) species formed by the mitochondrial NADPH oxidase complex activity [41] and/or other mechanisms [42], PARP1 will be activated, with a greater consumption and hence depletion of NAD^+^ [43,44].

### Metabolites of the transamination pathway

IPA possesses a range of pharmacological properties, including sedation, analgesia, sleep promotion and antioxidant and anticonvulsant actions, some of which may involve serotonin and melatonin [45,46]. It also possesses anti-inflammatory properties, best exemplified by its ability to suppress experimental colitis in mice and simultaneously, among other effects, to attenuate the expression of genes encoding Th1 cytokines and to enhance IL-10 gene expression in the colon [47]. These effects of IPA are mediated by the AhR, thus providing an example of the beneficial effect of activation of this receptor by endogenous ligands. However, as will be discussed below, over- or sustained activation of the AhR can cause harm. As stated above, the KP metabolite KA can also be formed from IPA. While both IPA and KA are endogenous AhR ligands, KA possesses both anti- and proinflammatory properties, hence its description as ‘Janus-faced’ [48].

### Metabolites of the decarboxylation pathway

Tryptamine possesses properties consistent with being a neurotransmitter and a modulator of monoamine function [49]. The formation of both IPA and tryptamine in the above two pathways is largely dependent on Trp levels. As is the case with IPA, tryptamine also activates the AhR, via a metabolite(s) produced following its oxidative deamination [50]. These latter authors and others [51] proposed FICZ as the potential effector AhR ligand, with an extremely high binding affinity. As stated above, FICZ, originally identified as a product of Trp photo-oxidation, can be formed metabolically by condensation of two molecules of indol-3-ylacetaldehyde or at least one molecule plus the α-hydroxylated metabolite of the other [10].

### Tryptophan metabolites produced by gut microbiota

Indole, a powerful AhR ligand [29] and the major bacterial metabolite of Trp [52] exerts a wide range of protective effects in the gastrointestinal tract including, among others, acting as a signaling molecule regulating bacterial motility, antibiotic resistance, antagonism of host cell invasion by virulent bacteria, ameliorating intestinal inflammation, suppressing production of proinflammatory chemokines and increasing that of anti-inflammatory cytokines (see [52] and references cite therein). Another protective metabolite is IPrA. It possesses anti-inflammatory, anti-oxidative and other protective properties [53–57].

Other AhR ligands are indol-3-ylacetaldehyde, indol-3-ylacetic acid and indol-3-ylaldehyde (I3A) [58]. I3A exerts its protective effect on intestinal mucosa via IL-22 [58], suppresses the expression of the proinflammatory cytokines IL-1β and IL-6 [59] and may have a beneficial value in protecting the lung against inflammation [32].

Indoxyl sulfate, generated mutually by microbes and host, is a potent AhR agonist [60]. At 1 µM, it activates it to the same extent as that achieved by a 10 nM concentration of the potent exogenous ligand TCDD. At 10 nM, indoxyl sulfate is half as effective as a similar concentration of TCDD. Indoxyl sulfate exerts harmful effects at various levels, including renal dysfunction and toxicity, vascular endothelial dysfunction and oxidative stress [60].

### Metabolites of the hydroxylation pathway

Serotonin exerts a wide range of effects in both the brain and periphery [61,62]. Its central effects have implications for disorders including alcoholism, anxiety, affective disorders, drug dependence, obsessive-compulsive disorder and appetite and sleep disorders. In the periphery, serotonin influences a range of gastrointestinal activities, including, among others, motility, peristalsis and secretions, and also influences cardiovascular and immune functions. The serotonin effects are mediated by various serotonin receptors and their subtypes, the serotonin transporter, and by covalent binding to different effector proteins [61]. Serotonin and its receptors exist in immune cells and evidence suggests that they modulate immune function and that serotonin–immune interactions can impact the behavioral response to inflammation [62,63]. Examples of the immune effects of serotonin include suppression of the release of IL-1β and TNF-α in peripheral blood cells and activation of T-cells ([61] and references cited therein). TPH isoform 1 is responsible for Trp hydroxylation to 5-HTP in the periphery.

Melatonin exerts effects on physiological processes, mainly the circadian rhythm and sleep [62], but also possesses antiinflammatory [64,65] and immunomodulatory [66] properties. The antioxidant property of melatonin appears to be superseded by its precursor N-acetyl serotonin [67]. In their review of previous studies of the immune-related effects of melatonin, Cho et al. [66] noted that it lowers levels of IL-1, IL-6 and IL-8 but not TNF-α.

### Metabolites of the oxidative (kynurenine) pathway

The KP produces a range of biologically active metabolites that impact many physiological processes and body systems, including protein synthesis and lipid metabolism (Trp), immune system (Kyn, 3-HK, 3-HAA, KA, AA, QA, PA, XA), carbohydrate metabolism (XA, QA, PA), nervous system (KA, QA) (see [1] for a review). The end product of the KP, NAD^+^, is a vital cellular effector at many levels, including energy metabolism and redox homeostasis, DNA repair, cellular stemness, immune function and molecular signalling [68]. [NAD^+^] is therefore critical for a wide range of cellular processes. NAD^+^ can be hydrolysed by NADase (NAD^+^ glycohydrolase) to nicotinamide or utilised (along with its phosphorylated derivative NADP^+^) as cofactor in various redox reactions or as substrate by a number of enzymes, including PARPs, SIRTs and NAD glycohydrolases. Activation of these latter three types of enzymes results in NAD^+^ depletion with potential harmful consequences.

Several Trp metabolites of the KP possess immunomodulatory properties. Of these PA and KA possess antiinflammatory properties, whereas others such as Kyn, 3-HK, 3-HAA, and QA are proinflammatory. KA also possesses proinflammatory properties (see above). A major feature in relation to inflammation is immunosuppression by Kyn metabolites, notably 3-HK, 3-HAA and QA suppressing allogeneic T-cell proliferation and undermining Th1 helper cells ([69,70]; see also [71] and references cited therein). At 10 µM, apoptosis of thymocytes by KP metabolites was strongest with 3-HAA > QA> 3-HK, with Kyn and AA exerting no significant effect [70]. T-cell proliferation is inhibited only by a large [Kyn] (IC_50_ = 157 µM) [69].

It is clear from the above account that a range of metabolites of Trp arising from its degradative pathways can impact immune function at multiple levels and, as described below, are targeted by tumours.

## Tryptophan metabolism in cancer

The KP is the most important pathway in relation to cancer biology. Various enzymes of the KP are widely distributed throughout normal tissues [72,73]. Notably, the following are enzymes most highly expressed in certain tissues: TDO2 (liver), IDO2 (liver, thyroid and testis), IDO1 (lung), FAMID (liver and kidney), KMO (liver), KYNU (liver), 3-HAAO (liver) and ACMSD (liver, kidney and synovial). Lesser or no expression of these enzymes occurs in other tissues. While production of KP metabolites is determined primarily by tissue distribution of these enzymes, these metabolites can be transported to various tissues by circulating blood for further metabolism by the expressed enzymes. It should be emphasized that gene expression of enzymes or increased enzyme protein are not always synonymous with enhanced enzyme activity. Most studies of gene expression of Trp metabolising enzymes in cancer are not accompanied by measurement of enzyme activities or plasma metabolites. Consequently, only measurement of enzyme activity directly or through changes in substrates and/or reaction products can provide the ultimate proof. In this regard, the widely reported decrease in plasma [Trp] with the concomitant increases in [Kyn] and the [Kyn]/[Trp] ratio, and the many preclinical studies and clinical trials of IDO/TDO inhibitors strongly suggest that activities of these two rate-limiting KP enzymes are increased in various cancer types. Moreover, the increase in plasma [Kyn] in patients with cancer can also result from an additional increase in the flux of plasma free Trp down the KP and also a potential inhibition (down-regulation) of KMO, as occurs in many cancer types [73]: all being determinants of the [Kyn]/[Trp] ratio [5]. Up-regulation of Trp transporters that also transport Kyn (see below) can further contribute to the Kyn elevation in tumours. Only a combination of these various determinants can explain the increases in the Kyn transamination product KA (by as much as 3-fold) in plasma, tumours, urine and intestinal mucus of patients with various cancer types (see [74] and references cited therein). It is, therefore, reasonable to suggest that KAT activity is also enhanced in some cancer types. Because of the high *K*_m_ of KAT for its Kyn substrate [3], KA formation requires an increase in [Kyn]. Whereas normal plasma [Kyn] varies between 1 and 3 µM, tumours can accumulate much larger amounts, e.g. 37–43 µM [24,75]. In the latter study [75] in female mice bearing CT26 tumours, tumoral [Trp] was also high (∼270 µM), compared with ∼130 µM in plasma. While tumours recruit Trp for their growth and proliferation through up-regulation of Trp transporters this huge rise in [Trp] concomitant with the Kyn elevation is most likely the result, at least in part, of increased availability and hence flux of plasma free Trp.

**Figure 4:**
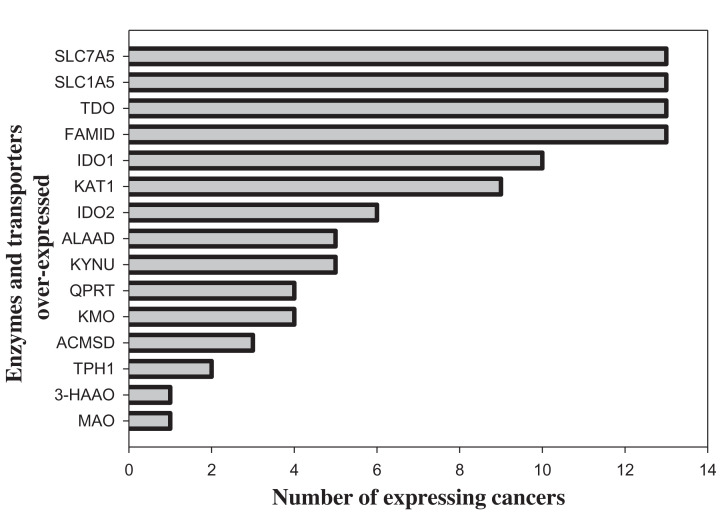
Numbers of cancer types expressing genes of enzymes of tryptophan metabolism and tryptophan transporters singly and in combinations Calculated from 28 cancer types as illustrated in Figure 4 in [73] by Perez-Castro, L., Garcia, R., Venkateswaran, N., Barnes, S. and Conacci-Sorrell, M. (2021) Tryptophan and its metabolites in normal physiology and cancer etiology. *FEBS J*. 2021: doi:10.1111/febs.16245. For enzyme abbreviations, see the text.

The KP enzymes most studied in cancer are IDO1, TDO2 and IDO2. IDO1 is up-regulated in a range of cancers, in particular, cervical cancer, diffuse large B-cell lymphoma, endometrial, head and neck squamous cell, lung and ovarian carcinomas [72]. IDO1 is also expressed in other cancers but at much reduced levels. TDO2 is particularly expressed to a high level in hepatocellular carcinoma (HCC) but to a much reduced extent in many other cancers [72]. IDO2 is expressed in fewer cancers, mainly those also expressing IDO1 [73], though additionally in colon, non-small-cell, pancreatic and renal cancers [76]. Perez-Castro et al. [73] established a comprehensive heat map binary analysis of gene changes in cancerous and adjacent normal tissue from the cancer gene atlas (TCGA), which revealed various changes in genes of Trp-metabolising enzymes and Trp transporters. In the study by these latter authors, TDO2 in HCC is, however, down-regulated, in contrast with the opposite report by van Baren and Van den Eynde [72] using data from the genome tissue expression (GTEx) project. The earlier up-regulation of TDO2 in HCC [72] was further confirmed [77] in tissue samples. These latter authors also reported TDO2 expression in intratumoral pericytes of most cancers and confirmed the absence of co-expression of IDO1 and TDO2 in glioblastoma that can be deduced from other data [73]. The reported [73] up-regulation of genes of the KP, the serotonin pathway and Trp transporters is summarised in Figure 4, which shows that genes of Trp transporters, TDO2 and FAMID are the most expressed in equal numbers of 13/28 cancer types. This suggests that tumours recruit not only the first key enzymes of the KP but also the transporters of their Trp substrate. Which of these two recruitment processes precedes the other is an important question that is discussed below.

Table 2, derived from the report by Perez-Castro et al. [73], lists the numbers of cancers expressing IDO2, IDO1 and TDO2 genes in conjunction with one another and with genes of FAMID, KAT1 and the Trp transporters SLC5A1 and SLC7A1. Of the 10 IDO1 expressing cancers, only 6 co-express TDO2, 3 co-express FAMID and 4 co-express each of the two Trp transporters, with ratios of 60%, 30%, 40% and 40% respectively. By contrast, of the 13 TDO2-overexpressing cancers, 6 co-express IDO1 (46%), 10 co-express FAMID (77%) and 9 (69%) co-express each of the two Trp transporters. Only 6 cancers were associated with expression of IDO2, 5 of which were associated with expression of the 2 Trp transporters, IDO1 and TDO2, and 2 with FAMID. The two Trp transporters were simultaneously expressed in 10/13 cancers (77%). KAT 1 (referred to as CCBL1) [73] was expressed in 9 cancers, only 2 of which were associated with IDO2 and IDO1, whereas 5–8 were associated with the other 4 parameters in Table 2 here. The greater association of KAT 1 with the Trp transporters and TDO2 and FAMID further emphasises the importance of KA production for tumours.

**Table 2:** Numbers of cancers expressing singly and jointly kynurenine pathway enzymes and tryptophan transporters

→	Alone	IDO2	IDO1	TDO2	FAMID	KAT1	SLC1A5	SLC7A5
↓								
IDO2	6	-	5	5	2	2	5	5
								
IDO1	10	5	-	6	3	2	4	4
								
TDO2	13	4	6	-	10	5	9	9
								
FAMID	13	2	3	10	-	6	9	9
								
KAT1	9	2	2	5	6	-	7	8
								
SLC1A5	13	5	4	9	9	7	-	10
								
SLC7A5	13	5	4	9	9	8	10	-

It would appear that tumours influencing Trp metabolism establish a strategy targeting the KP and aimed to maximise the production of certain Kyn metabolites. This involves (1) up-regulating TDO2, IDO1 and IDO2 in this order of preference; (2) expressing two Trp transporters to provide and ensure continued availability of the substrate of the above enzymes; (3) expressing the next enzyme FAMID to ensure that Kyn production is not hampered; (4) by expressing KAT1, tumours appear to favour the production of KA and while KA possesses both anti- and pro-inflammatory properties, it is likely that tumours intend to take advantage of these latter properties. Tumoral attempts to ‘high-jack’ the KP do not end here, but involve additionally [73] a significant down-regulation of genes of enzymes of serotonin synthesis, namely TPH1 and DDC (ALAAD) in 10 and 16 cancers that express TDO2 and IDO1 respectively, thus ensuring that Trp is not used by the serotonin pathway and avoiding the anti-cancer effects of metabolites of this pathway. Monoamine oxidase A (MAOA), the enzyme isoform specific for serotonin degradation, is however also down-regulated ín 17/28 cancers and is only significantly up-regulated in kidney renal clear cell carcinoma. The significance of MAOA down-regulation is currently unclear.

Other than KAT1 (CCBL1) being up-regulated in the 9/28 cancers listed by Perez-Castro et al. [73], other KP enzymes subsequent to FAMID are up-regulated in fewer cancers as follows: KYNU (5), KMO and QPRT (4 each), ACMSD (3) and 3-HAAO (1). Up-regulation of these enzyme genes was preferentially associated with that of genes of FAMID and TDO2, rather than with that of IDO1, with corresponding relative numbers of co-expressions for IDO1, FAMID and TDO2 being as follows: KAT 1 (2:6:6), kynureninase (3:2:4), QPRT (1:4:4) and ACMSD (0:3:3). Only KMO was equally co-expressed with these 3 genes in 3 cancers each, whereas 3-HAAO was co-expressed in 1 cancer only with IDO1 and TDO2. Up-regulation of KAT1 and KYNU ensures adequate production of KA and 3-HAA, respectively. It should be emphasized that human KYNU favours the 3-HK → 3-HAA, over the Kyn → AA reaction because of the greater affinity of the former reaction to its 3-HK substrate (*K*_m:_ 77 µM for 3-HK vs 1000 µM for Kyn). Although the low up-regulation of KMO implies a limited 3-HK formation, it is more likely that 3-HK production from Kyn will not be impaired. This may be due to the low *K*_m_ of KMO for Kyn (15–25 µM) [3]. Similarly, the down regulation of 3-HAAO in 19/28 cancers is unlikely to result in impaired formation of QA, as the *K*_m_ of the enzyme for 3-HAA is even much lower (2–3.6 µM) [3] and especially if ACMSD is expressed in only three cancers (in association with expression of FAMID and TDO2 [73]). However, alternatively, down-regulation of 3-HAAO can result in 3-HAA accumulation in favour of tumours. QPRT is also expressed in 4/28 cancers, all in association with expression of TDO2 and FAMID and 1 with IDO, and in other cancer types [78]. Its down-regulation in some cancers can not only lead to QA accumulation but also result in decreased conversion of QA to NAD^+^ via the *de novo* pathway. This can be doubly disadvantageous to host but profitable to tumours.

Less is known regarding enzymes of NAD^+^ metabolism via the Preiss-Handler and salvage pathways in cancer. NAD^+^ is vital for tumour proliferation and survival, acting at multiple levels including enhancing glycolysis and serine synthesis and activating PARPs and SIRTS [79,80]. The first enzyme of NAD^+^ synthesis from nicotinamide, NMPRT (Figure 3), is up-regulated in a range of cancers ([79] and references cited therein), and the second enzyme, NMNAT, can be activated under certain conditions to increase tumoral NAD^+^. Targeting these two enzymes and PARPs and SIRTs for cancer therapy has therefore been proposed. Tumours can therefore ensure an adequate supply of NAD^+^ via the salvage pathway while blocking the *de novo* pathway from QA to the host’s disadvantage.

## How do tumours manipulate the kynurenine pathway to their advantage?

From the above accounts, it can be concluded that tumours utilise the KP to their advantage in many ways as outlined in Table 3 and described below.

**Table 3:** Tumoral strategies for manipulating the kynurenine pathway

	Action	Host	Tumour
1	↑ NEFA, ↓ Albumin	Free Trp↑, Total Trp -,↓	Trp ↑
2	↑ TDO2, IDO1, IDO2	Trp ↓, NFK ↑	Trp ↓, NFK ↑
3	↑ Trp transporters	Trp ↓	Trp ↑
4	↑ FAMID	Kyn ↑	Kyn ↑
5	↑ KAT1	KA ↑	KA ↑
6	↓ KMO	3-HK ↓	3-HK ↓
			
7	↓ KYNU	3-HAA ↓	3-HAA ↓
8	↓ 3-HAAO	QA ↓	QA ↓
9	↓ ACMSD	PA ↓ QA ↑	PA ↓ QA ↑
10	↓ QPRT	QA ↑	QA ↑
11	↑ NMPRT	NAD^+^ ↑	NAD^+^ ↑↑
12	↑ NMNAT	NAD^+^ ↑	NAD^+^ ↑↑
13	↑ PARPs	NAD^+^ ↓	NAD^+^ ↑
14	↑ SIRTs	NAD^+^ ↓	NAD^+^ ↑

### Release of plasma tryptophan from albumin-binding sites

Trp availability to tissues and tumours is determined in part by extent of binding of the amino acid to plasma albumin, which is controlled by circulating [albumin] and [NEFA]. Decreased albumin and increased NEFA are both features of cancer and plasma free [Trp] is therefore likely to be elevated in cancer patients [81]. Thus, increases in free Trp of up to 135% occur in various cancers [82–84] and total [Trp] is decreased in at least seven types of cancer by 13–22% and by 34–48% in tumour-bearing rats ([81] and references cited therein). The free Trp elevation is due to a decrease in albumin and an increase in NEFA levels. For example, Namendys-Silva et al. [85] reported decreases in plasma [albumin] below 35 g/L (the lowest value in the 35–50 g/L normal range) in 82% of a 200-patient cohort, with an average for the whole group of 18 g/L. Several studies have also demonstrated elevated [NEFA] in cancer patients [84] and experimental cancer models [84,86,87]. Increased Free Trp availability is a major factor in determining the flux of free Trp down the KP in rats and humans [26,88–90], and it is therefore likely that the flux of free Trp down the KP in patients with cancer will be enhanced irrespective of whether TDO2 or IDOs are activated. In tumours, free Trp taken up from host can progress to Kyn metabolites as long as the necessary enzymes are expressed.

### Up-regulation of tryptophan transporters by tumours

This is likely to be of major importance for tumour growth and survival independently of KP manipulation, as Trp along with other essential amino acids are obligatory requirements for tumour growth and development. Tumours recruit amino acids by up-regulating amino acid transporters [73,91–97]. In their review article, Perez-Castro et al. [73] focused on two Trp transporters: SLC1A5 and SLC7A5, both of which are up-regulated in 13 cancer types and in 9/13 cancers in which TDO2 is also up-regulated, in 9/12 cancers expressing FAMID, but only in 4/10 cancers expressing IDO1. These latter authors focused on these two transporters in part because their co-expression correlates with poor outcome in a range of cancer types, but not if co-expressed with IDO1: perhaps this explains the small number of IDO1-expressing tumours in which the two transporters co-associate. It would seem that tumours do not gain much by associating transporters with IDO1. Could this be because Trp breakdown to Kyn metabolites is much more likely to be enhanced by associating transporters with TDO2, whose capacity for Trp is far greater than that of IDO1 (*K*_m_ = 190 vs 20–50 µM) [3]?

Another Trp transporter, SLC6A14 (also known as (ATB^0,+^) is a concentrative transporter, in contrast with the above two exchange ones, and therefore has a much greater capacity for transporting Trp into tumours, and is up-regulated in a range of cancers of epithelial origin [92,93,96]. Its concentrative nature and the broad spectrum of amino acids, including the essential ones, it transports make SLC6A14 the ideal transporter for tumour growth and development [94]. A novel amino acid transporter that accounts for 50% of Trp uptake has been reported [98] to be expressed by IDO-positive tumour cells. These authors reported that this transporter is biochemically different from the L transporter system in many ways and, under conditions of low Trp, it significantly increases Trp entry into tumours. Earlier, Seymour et al. [99] reported a high-affinity Trp-selective amino acid transporter in human macrophages. Its Trp-transporting activity is 100-fold higher than that of the L system, with a *K*_m_ of 300 nM. Future studies should consider expression of these Trp-selective transporters. That tumours can accumulate much Trp has been demonstrated, e.g. in colon and stomach tissues of patients with cancer, [Trp] rises to 40–70 µM, reflecting 1.5- to 2.3-fold increases [100] and in a mouse model of colon carcinoma, tumour tissue [Trp] is 270 µM, compared with 130 µM in plasma [75].

### Up-regulation of KP enzymes (IDO1, IDO2, TDO2, FAMID and KAT1)

To facilitate production of Kyn metabolites, as well as by increasing Trp uptake, tumours express KP enzymes, mainly the first and rate-limiting IDO and TDO, but also certain subsequent enzymes, but to varying extents, as depicted in Figure 4. Whereas IDO2 is expressed in a few cancers (four-to-six), both IDO1 and TDO2 show a much broader expression [72,73,101]. The equally broader expression of FAMID [73] ensures adequate production of Kyn.

Investigators have focused on Kyn as the major product of KP activity by tumours, as little was known about changes in subsequent KP enzymes. Studies then focused on measuring IDO1 mRNA expression and determination of the [Kyn]/[Trp] ratio as an indirect measure of enzyme activity. However, this ratio is not exclusive to IDO but can also be altered by a variety of determinants, namely TDO activity, flux of plasma free Trp down the KP and activity of the subsequent KP enzymes KMO and KYNU [102]. The role of Kyn was examined mainly in relation to activation of the AhR, modulation of reactive oxygen species and inflammation (see, e.g., [24,103,104]. However, Kyn is not a strong AhR activator. Its activity in this regard is superseded by that of its transamination metabolite KA, whose AhR activation is the highest among KP intermediates, followed by XA, with Kyn being the least active. The role of KA (and KAT) in AhR activation merits investigation in future cancer-related studies for a number of reasons. First, it is no surprise that KAT 1 (also known as CCBL1) is also significantly up-regulated in nine cancer types [104], 1 short of cancers expressing IDO1 and 3-4 short of those expressing FAMID and TDO2 (Table 2). Second, even if KAT is not activated, the KAT reaction of Kyn → KA can simply proceed to fruition if [Kyn] is high enough because of the high *K*_m_ of the enzyme for Kyn (0.96–4.7 µM) [3]. Third, the recently reported [28] novel metabolic immune checkpoint IL-4I1 activates the AhR via KA and the Trp transamination metabolite IPA, and not via Kyn. IL-4I1 increases [KA], but not [Kyn], and KA can also be formed from IPA as described above.

### Down-regulation of other KP enzymes (KMO, KYNU, 3-HAAO and ACMSD)

Whereas KP enzymes preceding KMO, and also the 2 Trp transporters SLC1A5 and SLC7A5, are up-regulated in 32-46% of cancer types, KMO and subsequent enzymes show lesser expression, ranging from 3 to 18% (Figure 4). It appears that the majority of tumours down-regulate these latter enzymes to maximise Kyn and KA formation, and while their down-regulation of KYNU and 3-HAAO decreases QA production, QA levels could still be raised by down-regulation of QPRT and ACMSD. This appears to be clever dual manoeuvring by tumours to simultaneously maximise AhR activation and undermine T-cell immunity.

### Tumoral requirements for NAD^+^ (up-regulation of NMPRT and NMNAT)

NAD^+^ is vital for tumour cell proliferation at various levels: rapid ATP generation through the preferred glycolytic pathway, decreased ROS production by avoiding aerobic conditions, and generation of purine and pyrimidine nucleotides to support DNA replication and RNA production through activation of the pentose phosphate pathway [68,79]. Enhancing NAD^+^ availability is therefore a further tumoral manoeuvre which it achieves by up-regulating nicotinamide phosphoribosyltransferase (NMPRT) in the salvage pathway (Figure 3). NMPRT is up-regulated in a wide range of cancer types and cancer cell lines [68,105]. Although NAD^+^ can also be synthesised from nicotinic acid in the Preiss-Handler pathway, many cancers do not up-regulate nicotinate phosphoribosyltransferase (NPRT) [79] and must therefore rely solely on NMPRT up-regulation. Nicotinamide mononucleotide adenylyltransferase (NMNAT) (Figure 3) could also be a potential tumoral target, as it is expressed in colorectal cancer [106] and neuroblastoma [107]. Tumours also modulate activities of NAD^+^-consuming and degrading enzymes as outlined below. It is of additional interest that up-regulation of NMPRT induces SIRT1 expression [68].

### Up-regulation of NAD^+^-consuming enzymes (PARPs and SIRTs)

Because PARP1 and SIRT1 are the largest consumers of NAD^+^, the following brief account will be limited to these two members of their families. PARP activation is observed in a range of cancers and can be promoted by irradiation and in fibroblasts by DNA alkylating agents or by H_2_O_2_ leading to necrotic cell death [108,109]. Virus-related pulmonary diseases are also associated with PARP activation (see [110] and references cited therein). An important difference between PARP activation in cancer and that in pulmonary disease is that it is accompanied in tumours with up-regulation of NMPRT and also NMNAT, such that nicotinamide is continually recycled to NAD^+^, further enhancing PARP activity and providing NAD^+^ to the tumour in perpetuity. PARP activation is, however, generally protective, as it can produce an anti-inflammatory response. Thus, whereas PARP activation can be harmful in a number of cancer types (breast, gastric and small-cell lung cancers and melanoma), it is beneficial in pancreatic cancer [105]. Similarly, sirtuins 1-7 can also act to suppress or promote tumours. Thus, both SIRT1 and SIRT2 are activated in cancers, acting by diminishing tumour suppressors and stabilising oncogenes, or by activating glucose-6-phosphate dehydrogenase to increase NADPH production for tumour cell proliferation [105], but SIRT3 and SIRT4 can also protect cells against death by maintaining mitochondrial NAD^+^ levels following stress ([111]; see also the detailed discussion in [112]). In regard to proliferation, intracellular compartmentation of NAD^+^ becomes an important issue, as the cytosolic compartment is where glycolysis and the pentose phosphate pathway exist, and it is of interest that both NMPRT and NAPRT are located in the cytosol [113].

### Tryptophan disposition in cancer

The above account illustrates how tumours establish suitable conditions for their growth and proliferation by increasing their uptake of tryptophan and ensuring an adequate supply of NAD^+^ and by simultaneously undermining host defences through a series of effects involving increased Kyn and KA, AhR activation, decreased NAD^+^ synthesis from Trp in the *de novo* pathway and up-regulating NAD^+^-consuming enzymes. Tumours also further undermine immune defences by manipulating Trp in their microenvironment to their own advantage. This is achieved by depleting their Trp content to an extent incompatible with T-cell survival. T-cell survival requires a minimal [Trp] of 10 µM, as their proliferation is inhibited at lower [Trp], with 1 µM causing complete inhibition [114]. When tumoral [Trp] falls to 5 µM or below, SLC1A5 is up-regulated, but this up-regulation ceases if [Trp] rises to 10 µM or above [115]. As stated above, IDO tumour cells depleted of Trp express a Trp-specific transporter which accounts for ∼50% of Trp uptake [97]. Thus, tumoral [Trp] is critical for both T-cell and tumour proliferation, with determinants such as IDO, TDO, Trp transporters and plasma Trp availability all playing important roles in tumour biology. A tumoral [Trp] ≥ 10 µM would be an ideal therapeutic target.

## Tumoral immune escape and how to defeat it by modulation of tryptophan metabolism and disposition

Tumours exploit all available means to escape the host's immune defences. Apart from up-regulating the above determinants related to Trp metabolism, various mechanisms of tumoral immune escape have been proposed, including (1) metabolic stress [116], (2) hypoxia-induced NAD^+^ intervention [111], (3) M2 macrophage-derived exosomal microRNA-155-5p down-regulating ZC3H12B [117], (4) various mechanisms operating during the cancer immune cycle [118], (5) a role for cancer stem cells [119] (6) NAD^+^ metabolism to maintain inducible PD-L1 expression [120] and (7) non-coding RNAs [121,122]. A discussion of potential links of these mechanisms to Trp metabolism is outside the scope of this text, though the view that the KP has a footprint in every cancer has been expressed [123]. A recent example is the observation that Trp may enhance PD1 blockade [124]. Indeed, a wide range of studies and clinical trials have been performed to modulate KP enzyme activities as cancer therapeutic strategies, mainly related to IDO1, TDO2, KAT and KYNU, that are too numerous to list here, but the following are useful reviews and accounts [75,101,125–128]. Other Trp-degradative pathway enzymes have also been targeted, but to a much lesser extent, e.g., TPH1 inhibition for carcinoid syndrome symptoms [129] with telotristat etiprate being a promising therapy [130] and MAO1 inhibition for prostate cancer [131]. Gut microbiota acting in part through Trp metabolites may also play a role in some cancers, e.g., brain tumours and colorectal cancer [132–134]. No attempt will be made here to discuss potential pharmacotherapies involving KP modulation, as these are discussed at length in some of the above references and elsewhere. Based on the above accounts, several strategies based on KP activity and related processes should be adopted to combat tumoral immune escape. Combination therapy is very likely to become the best approach. A number of aspects paving the way to adopting such strategies are briefly described below and illustrated in Figure 6 further below.

### Inhibition of Trp transport to tumours

Undermining Trp transport to tumours should be the initial top priority in combating immune escape, as tumours have to grow and take hold before, or concurrently with, starting to evade immune defences. The time-course of expression of Trp-specific and –non-specific amino acid transporters and IDO/TDO by tumours requires investigation. Current evidence from studies in placental villous explants [114] suggests that, whereas IDO is already expressed, inhibition of Trp transport decreases its activity. The study by Silk et al. [98] shows that IDO1 induction leading to Trp depletion precedes up-regulation of a Trp transporter. Whether a similar situation exists with TDO2 up-regulation remains to be established, although the greater co-expression of Trp transporters with TDO (Table 2) suggests that this may also be the case. It is further reasonable to speculate that inhibition of Trp transport to tumours makes their up-regulation of IDO/TDO redundant, but necessary, as it could be viewed as tumours conserving the little Trp they have for their survival and simultaneously depriving T-cells of an essential nutrient. Blockade of Trp transporters, e.g., SLC6A14, is therefore one antitumour strategy [96]. Several potential SLC inhibitors have been developed, but this particular area of pharmacological research and development is relatively at its infancy and many factors play important roles in the process, including structural biology, membrane protein expression, and inhibitor screening methodology [73,135]. Amino acid anti-cancer prodrugs transported into tumours via the above and other Trp transporters may provide another strategy [93].

**Figure 5:**
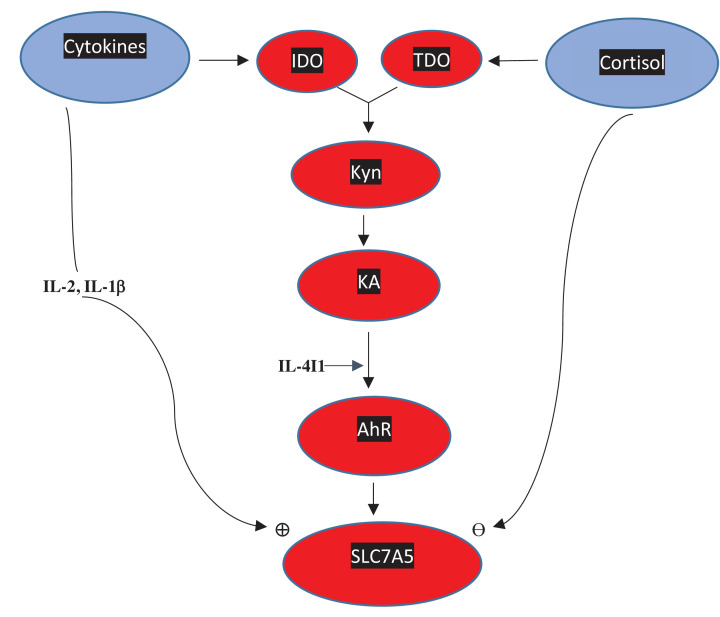
Proposed IDO/TDO-AhR-SLC7A5 axis Abbrevations: AhR, aryl hydrocarbon receptor; IDO, indoleamine 2,3-dioxygenase; IL, interleukin; KA, kynurenic acid; Kyn, kynurenine; SLC7A5, solute carrier transporter 7A5; TDO, tryptophan 2,3-dioxygenase. Induction of IDO by proinflammatory cytokines and/or of TDO by cortisol activates the kynurenine pathway leading to increased production of kynurenine and its consequent transamination to kynurenic acid. KA activates the AhR, which in turn up-regulates the neutral amino acid transporter SLC7A5. Two interleukins are known to enhance expression of this transporter, whereas cortisol may exert the opposite effect.

The special role of Trp transporters in cancer biology raises further important questions that merit exploration here. As transporters including SLC7A5 also transport Kyn, they can contribute further to its influx into tumours, thereby bypassing IDO, TDO and FAMID, activating the AhR, further enhancing IDO expression, and undermining invading T-cell function [96,136]. IDO activity, on the other hand, enhances Trp uptake into tumours by inducing the Trp transporter responsible for 50% of Trp uptake [98] and, whereas these latter authors [98] suggested that the stimulus for expression of this transporter is the Trp depletion induced by IDO, evidence exists for up-regulation of SLC7A5 by the AhR [137–139] secondarily to IDO induction, thereby representing a positive feed-forward mechanism for AhR activity [140], as illustrated in the proposed IDO/TDO-AhR-SLC7A5 axis in Figure 5 here. The complexity and some paradoxes of the interactions between the parameters listed are exemplified by the observations that SLC7A5 is up-regulated by both IL-1β [141–143] and IL-2 [144] and that glucocorticoids inhibit SLC7A5 expression, as suggested by the effects of prednisolone [142] and dexamethasone [145]. The question of competition between Kyn and Trp for entry into microenvironments was raised by Sinclair et al. [136], who suggested that Kyn is unlikely to compete with Trp for uptake by T-cells given the 10-fold difference in their SLC7A5 affinities (*K*_m_ for Kyn: ∼200 µM vs Trp ∼20 µM). These authors [136] however alluded to a possible scenario of fierce nutrient competition wherein Kyn could compete. One such scenario could be visualised in the tumour microenvironment, wherein extremely low [Trp] coupled with high [Kyn] following IDO/TDO up-regulation could occur. In fact, given that Trp depletion following IDO or TDO induction *in vivo* and not in closed *in vitro* culture systems never reaches >60% of plasma values (averaged 60 µM) [5] and the huge rise in tumoral [Trp] in experimental *in vivo* models that is twice as high as plasma levels [75], it is more likely that the Trp depletion in the tumoral microenvironment to levels <10 µM is not the sole result of IDO/TDO induction, but could additionally involve a Kyn inhibition of Trp uptake.

**Figure 6:**
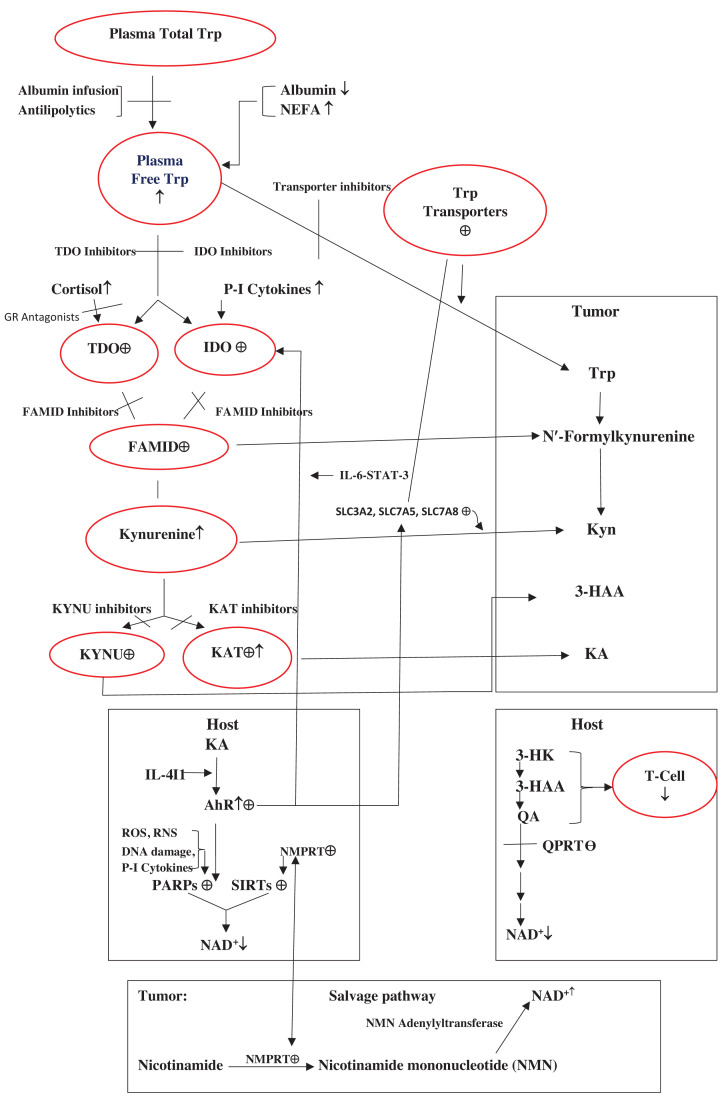
Graphic summary representation of tryptophan metabolism and disposition in cancer biology and proposed therapeutic strategies See the text for abbreviations. Plasma Trp exists largely bound to albumin and its tissue uptake depends on how much exists freely, which depends on levels of albumin and the physiological displacers NEFA. Albumin is decreased and NEFA are increased in cancer, hence the increase in free [Trp]. Kyn metabolite formation is controlled by flux of free Trp down the KP and activities of TDO and IDO. Increased production of some Kyn metabolites in host and tumour undermines innate immunity. Down regulation of QPRT in host results in impaired conversion of QA into NAD^+^. A further loss of NAD^+^ in host results from activation of the AhR by KA and subsequent up-regulation of the NAD^+^ consumers PARPs and SIRTs. Tumours recruit Trp from host with the help of Trp transporters and ensure an uninterrupted production of Kyn metabolites to undermine T-cell activity by up-regulating TDO, IDO and FAMID and simultaneously maintain adequate NAD^+^ levels by up-regulating the salvage pathway.

The proposed scheme in Figure 5 includes contributions of both IDO and TDO to the subsequent AhR activation. However, in cancer types in which SLC7A5 is co-expressed with TDO and not with IDO (colon and rectum adenocarcinomas, lung adenocarcinoma and lung squamous cell carcinoma) [73], AhR activation is likely to involve only TDO activity. That TDO over-expression is a prognostic marker of overall survival and progression-free survival of patients with cancer has recently been suggested in a meta-analytical review [146].

SLC7A5 and other Trp transporters exert a range of effects on body systems apart from amino acid transport, including control of m-TOR signaling and glycolysis, and inhibition or silencing of SLC7A5 exerts deleterious effects across a wide range of physiological processes. Little is however known about potential effects of SLC7A5 blockade on Trp metabolism, other than inhibition of cellular Trp uptake. Inhibition of SLC7A5-mediated amino acid influx by BCH (2-amino-2-norbornanecarboxylic acid) or siRNA knockdown leads to a marked reduction of IL-1β production by monocytes [143], thereby possibly inhibiting IDO induction by this cytokine. The above account clearly demonstrates the need for further studies of the role of Trp transporters in cancer biology and therapy.

### Inhibition of IDO1, TDO2 and IDO2

The next step is to target IDO1 and TDO2 (and also IDO2) with inhibitors. Evidence from previous studies [73,75] suggests that tumours prefer to up-regulate TDO2 more than IDO1 or IDO2. The widespread up-regulation of the glucocorticoid receptor in cancer types (see below) further emphasises the importance of TDO2 in cancer biology. A potential explanation of tumoral preference for TDO2 is that of TDO2 producing more Kyn and its metabolites in view of its greater capacity for Trp, compared with IDOs and also for its ability to cause a greater depletion of Trp in tumour microenvironment. Also, tumours may sense that [Trp] above 50 µM can inhibit IDO1 activity [147] by a mechanism involving reversed sequence of binding of Trp and O_2_ to the enzyme [148]. The latter authors estimated a Trp *K*_i_ for human IDO1 of 65–68 µM and it is therefore of interest that, in the limited studies in patients, tumoral [Trp] does not exceed 40–70 µM (see above). The Trp inhibition of human IDO1 is underpinned by the presence of an inhibitory substrate-binding site (si) that is absent from TDO2 [149]. Therapeutic targeting of IDO or TDO will depend on which enzyme(s) is up-regulated, but co-expression of these enzymes as outlined above justifies the use of joint or dual TDO2/IDO1 inhibitors. It is possible that, in cancer types associated with IDO1 up-regulation, the use of IDO1 inhibitors may ‘force’ tumours to up-regulate TDO2: a possibility worthy of investigation. Over expression of IDO2 and its wide range of effects in cancer also justifies targeting this isoform [74]. Another advantage of TDO/IDO inhibition in cancer therapy is that of the resultant elevation of tumoral [Trp] blocking Trp transport into tumours and enhancing T-cell immune function and anti-tumour activity.

In developing IDO1/TDO2 inhibitors, a number of aspects should be considered, as detailed in [150]. These include structure-based considerations, kinetic factors (substrate and cofactor concentrations, competition with O_2_ and/or Trp), analytical measurements (product elevation or substrate depletion), docking studies and unforseen effects of functional groups.

### Inhibition of FAMID

Little is known of targeting FAMID, except that its inhibition can prevent the progress of Trp down the KP and it is of interest that FAMID is co-expressed mainly with TDO2, the 2 Trp transporters and KAT1, but minimally with IDOs (Table 2). As far as is known, no genetic abnormality in FAMID has been recognised in diseases. The molecular structure of the enzyme and its catalytic mechanism have been studied [151] and can be used in drug developmental efforts targeting the enzyme for therapeutic purposes. Drugs based on the structure of the powerful FAMID inhibitory organophosphate insecticide diazinon could however induce a plasma Kyn elevation and increased transamination of 3-HK to XA [152].

### Inhibition of KMO, KAT and KYNU

By contrast with FAMID, KMO targeting has been widely studied for a variety of conditions, mainly those involving neuronal dysfunction. KMO inhibition could be regarded as a double-edged sword in relation to cancer. On the one hand, it can lead to accumulation of Kyn and consequent activation of the KAT reaction of Kyn → KA, thereby enhancing AhR activity to the advantage of tumours. On the other hand, KMO inhibition can limit production of 3-HK, 3-HAA and QA, which should promote T-cell anti-tumour immune defences. However, as stated above, tumours can compensate for the resulting NAD^+^ depletion by up-regulating NMPRT and NMNAT through the salvage pathway. In any case, few cancer types up-regulate KMO, as shown in [73], including triple negative breast cancer [153], and it is of interest that the latter authors reported near total absence of up-regulation of KAT II or KYNU in breast cancer databases. KAT I and KYNU are however up-regulated in 9 and 5 cancer types, respectively, but are co-expressed with KMO in only 1 or 2 cancer types [73]. KAT inhibition has been focused mainly on schizophrenia, wherein glutamatergic hypoactivity is an important feature. However, inhibition of KAT I or KAT II in cancer therapy is desirable as a way of limiting KA formation and hence AhR activation. These features of AhR activation and the role of the check-point IL4I1 further emphasise the need to focus future studies on the role of KA in cancer and on strategies aimed at lowering its levels, which are raised in tumours, sera, bone marrow and intestinal mucosal material of patients with cancer types [74].

### Inhibition of NMPRT and NMNAT

As well as facilitating QA conversion to NAD^+^, QPRT interacts with caspase-3, thereby inactivating it and preventing the occurrence otherwise of spontaneous cell death [78]. The down-regulation of QPRT in many cancer types helps tumours in one way but is compensated for in another by tumours acquiring most of their NAD^+^ via the salvage pathway [155] and by altering NAD^+^ compartmentation between mitochondria and cytosol [154,155]. NMPRT, the rate-limiting enzyme of the salvage pathway, is highly expressed in a wide range of cancer types and its levels correlate inversely with patient survival ([155] and references cited therein). These latter authors reported that many studies with NMPRT inhibitors have yielded positive results in *in vitro* and animal models but not in patients. NMNAT is another NAD^+^-synthesising enzyme that is up-regulated in some cancer types and evidence exists for involvement of an NMNAT2-SIRT3-NAD^+^ axis in cell proliferation [79].

### Inhibition of AhR activation and the NAD^+^-consuming enzymes PARPs and SIRTs

As stated above, KA and IPA are the most potent AhR ligands among Trp metabolites. Whereas the KP provides the bulk of KA, the Trp transamination pathway in host and gut microbiota supplies IPA. Gut microbiota Trp metabolism can however result in protection against, or promotion of, cancer types (see above and also [52,132–134]). Similarly, the role of AhR activation is also controversial. Thus, while the AhR is activated in hepatocellular carcinoma [156] and reported to enhance tumour growth, migration and metastasis [157] possibly acting via IDO induction [34], and is associated with malignant progression and poor survival of patients with brain tumours [24], others [158] reported that AhR activation by kynurenine impairs progression and metastasis of neuroblastoma. The AhR can therefore be viewed as both friend and foe [22].

An important feature of cancer biology is the identification [28] of IL-4I1 (*L*-Phe oxidase) as a metabolic immune checkpoint that activates the AhR and promotes tumour progression by enhancing cancer cell motility and suppressing adaptive immunity. It activates the AhR via KA and IPA, thus further emphasising the importance of these 2 Trp metabolites in future studies. Targeting IL-4I1 has been proposed by the above authors to combat tumoral immune escape. The authors proposed that IL-4I1 increases [KA] via IPA and that the latter is the result of Phe oxidase activity towards Trp. It appears from their graphic presentation [28] that Phe oxidase causes ∼66% depletion of culture medium [Trp]. This enzyme exists in mammals mainly in liver, kidney and to a lesser extent elsewhere [159]. The enzyme purified from *Pseudomonas* is less active towards Trp, exhibiting 9% of the activity towards Phe [160] and an approximate comparison from the graphic data of Sadek et al. [28] gives a corresponding value of ∼ 7.4%. The enzyme, generally referred to as *L*-amino acid oxidase (LAAO), has been widely studied in microorganisms and exhibits variable affinities and capacities for Phe, Trp and Tyr, as suggested by its *K*_m_ values being 0.011–31.55, 0.08–4.2 and 0.019–2.2 mM, respectively [7]. However, in mammalian (rat) kidney mitochondria, the enzyme shows equal activities with Trp and Phe as substrates, both of which represent 35 and 32%, respectively, relative to that of Leu [161]. In leukocytes, ÍL-4I1 (LAAO) is localised in lysosomes and exhibits an acidic pH preference [162]. In various snake species, the *K*_m_ of LAAO for Trp is much lower than that for Phe in some and moderately higher in others ([163] and references cited therein). Thus, in all probability, IL-4I1 is capable of producing sufficient IPA from Trp to enhance significantly the AhR. A number of IL-4I1 inhibitors are known, including anthranilic acid and p-aminobenzoic acid, the amino acids Ala, Arg, Cys, Leu, also riboflavin, quinine, chlorpromazine, indomethacin, aspirin and other compounds such as *L*-propargylglycin, aristocholic acid and suramin ([164] and references cited therein). N-Acetyl tryptophan and *o*-aminobenzoic acid also inhibit IL-4I1 activity, with *K*_i_ s of <130 and 12 µM, respectively [165]. Future studies may identify stronger inhibitors.

As the AhR controls PARP gene expression [28,36], the latter can be linked to Trp metabolism. Preclinical data suggest that PARP inhibitors may be effective therapies for inflammatory, metabolic and neurological disorders [166]. Thus, as stated above, PARP1 is up-regulated in cancers [109] and inhibition of PARP1 and other PARPs is currently one therapeutic strategy [167–170]. While PARP over activation can deplete NAD^+^ and ATP, its up-regulation in tumour microenvironment is necessary for tumour survival through DNA repair [109]. Also, as stated above, the nicotinamide arising from the PARP reaction in tumours can be recycled to NAD^+^ by the up-regulated NMPRT and NMNAT.

The other NAD^+^-consuming enzymes, the SIRTs, are also up-regulated in a range of cancer types, as they are also used by tumours to promote their cell proliferation and progression through mechanisms involving NMPRT regulation of SIRT1 activity and a positive feedback loop of c-Myc-NMPRT-SIRT1 ([68] and references cited therein). Targeting SIRT activity in cancer therapy has therefore been proposed [68,77]. Competition for NAD^+^ between SIRT1 and PARP1 is an important consideration [171].

Whereas DNA damage in cancer can trigger PARP up-regulation, it is likely that AhR activation will play a major role in enhancing PARP expression and targeting the AhR could be considered a priority approach in cancer therapy. AhR inhibitors have been shown in preclinical studies to reverse IDO/TDO-mediated tumour progression and to improve the efficacy of PD-1 blockade [172]. However, while many cancer cells are sensitive to AhR inhibition *in vitro*, clinical trials failed to demonstrate efficacy, possibly because of the broad range of AhR ligands and transcriptional targets ([73] and references cited therein).

### Other approaches (albumin infusions, antilipolytic agents and glucocorticoid antagonism)

To decrease plasma free Trp availability to tumours with or without inhibition of Trp transporters, albumin infusions and inhibition of lipolysis may be useful. Albumin infusions have been inappropriately prescribed in most patients with cancer [173]. Previous studies have indicated that plasma free [Trp] becomes significantly elevated when albumin levels are decreased by ≥19% [174]. A decrease in [albumin] over 19% would therefore justify albumin infusion, especially if accompanied by a NEFA elevation. Antilipolytic agents could also be considered, e.g., nicotinic acid. Glucocorticoid antagonists could also have a therapeutic role in lowering TDO induction by cortisol, whose circulating levels are raised in cancers ([173] and references cited therein). Glucocorticoid receptors are expressed in 20 solid tumour types [175] including most of those expressing TDO2 [73] and 9 other cancer types including hepatocellular carcinoma. In stress states, as would be the case in cancer, a new glucocorticoid receptor appears, as has been shown in rats under a number of stressful conditions including bearing an AH 130 tumour [176] that is specific for induction of TDO, but not of the other glucocorticoid-inducible enzyme Tyr aminotransferase [177]. Although glucocorticoids have been used in cancer patients to minimise side effects, they can promote immune escape, e.g., in lung and cervical tumours treated with antineoplastic agents and dexamethasone [178]. IDO1 is expressed in these two cancer types and an explanation of this deleterious effect of dexamethasone has been advanced in relation to interferon actions in COVID-19 infection [110], based on the ability of this steroid to potentiate the IFN-γ induction of IDO1 by prostaglandin PGE2, but to inhibit the corresponding IDO1 induction by IFN-α. Glucocorticoid antagonism is therefore a desirable strategy to undermine tumoral immune escape. That antagonists can be useful is suggested by enhancement of tumour cell apoptosis in chemotherapy-resistant triple-negative breast cancer cells by mifepristone [179] and reversal of docetaxel resistance in prostate cancer patients by RU-486 and cyproterone acetate [180]. Both RU-486 and mifepristone block glucocorticoid induction of TDO [181].

## Conclusions and comments

Tryptophan metabolism is an important feature of tumour biology and an immunotherapeutic strategy in cancer. Tumours induce profound changes in Trp metabolism and disposition that bolster their growth and proliferation and undermine the host’s immune defences. The kynurenine pathway is the primary focus of tumours and is cleverly manipulated in many ingenious ways. Targeting the kynurenine pathway in cancer therapy should start with blocking Trp transport into tumours by inhibition of Trp-specific and –unspecific transporters and other metabolic interventions, even before pharmacological inhibition of pathway enzymes. The possibility that inhibition of Trp transport may arrest tumoral expression of TDO/IDO, for which there is some evidence (see above), could be further explored in experimental tumour cell systems. As TDO appears to be up-regulated in more cancer types than has hitherto been assumed and appears to be a prognostic factor in patient survival, tumour tissues should be assessed for TDO2 in addition to IDO1 and IDO2 before embarking on mono or multiple therapies with inhibitors of these enzymes. The possibility that IDO inhibition may lead to tumoral expression of TDO that could explain the failure of IDO inhibitors in some clinical trials could be explored. N′-formylkynurenine formamidase (FAMID) is emerging as another up-regulated KP enzyme in cancer and targeting it could be explored. Addressing other mediators of tumour activity related to Trp metabolism, notably activation of the AhR, IL-4I1, PARPs and SIRTs will be equally important, with AhR inhibition being a priority. Kynurenic acid and indole-3-yl pyruvic acid should receive greater emphasis and their roles as AhR agonists in tumoral immune escape should be explored, at least until therapeutically effective AhR inhibitors are discovered. Exploring the role of gut microbiota in this context can yield important information. The greater association of expression of Trp transporters with that of TDO over IDO merits appraisal. If tumours can sense Trp depletion within their microenvironments, they may be able to sense basal Trp tissue levels and decide which enzyme (TDO/IDO) should be expressed. No information is available on human tissue [Trp], but a brief comparison of data available from studies [182–184] in rats suggests that [Trp] is highest in kidney > liver > intestine > spleen > lung > pancreas > brain. It is notable that TDO2 is highly expressed in cancers of the first three tissues. Finally, strategies to control and maintain tumoral [Trp] at > 10 µM should be explored. Could intra-tumoral injection of Trp become a viable strategy? Combination therapy is likely to be the best approach when targeting Trp metabolism.

## Abbreviations

ACMSD: 2-amino-3-carboxymuconic acid-6-semialdehyde decarboxylase
AhR: aryl hydrocarbon receptor
FAMID: N′-formylkynurenine formamidase
IDO1: indoleamine 2,3-dioxygenase 1
KAT1: Kyn aminotransferase 1
KMO: Kyn monooxygenase
KYNU: kynureninase
LAAO: L-amino acid oxidase
PARP: poly (ADP-ribose) polymerase
QPRT: quinolinate phosphoribosyltransferase
TDO2: Trp 2,3-dioxygenase
Trp: tryptophan
